# The Global Burden of Eustachian Tube Dysfunction in Older Patients: Pathophysiology, Clinical Consequences, Diagnostic Challenges and Therapeutic Perspectives—A SANRA-Based Study

**DOI:** 10.3390/healthcare14183001

**Published:** 2026-09-14

**Authors:** Giannicola Iannella, Pasquale Pio Caputo, Antonino Maniaci, Jerome R. Lechien, Giovanni Cammaroto, Carlos M. Chiesa-Estomba, Pierre Guarino, Giovanni Salzano, Luigi Angelo Vaira, Stéphane Gargula, Hilal Dincer D’Alessandro, Patrizia Mancini, Manuele Casale, Antonio Moffa, Elena Russo, Annalisa Pace, Giuseppe Magliulo, Armando De Virgilio

**Affiliations:** 1 Department of Sense Organs, Sapienza University of Rome, 00185 Rome, Italy; pasqualepio.caputo@uniroma1.it (P.P.C.); hilal.dincer@uniroma1.it (H.D.D.); p.mancini@uniroma1.it (P.M.); elena.russo@uniroma1.it (E.R.); annalisa.pace@uniroma1.it (A.P.); giuseppe.magliulo@uniroma1.it (G.M.); armando.devirgilio@uniroma1.it (A.D.V.); 2 Department of Medicine and Surgery, University of Enna Kore, 94100 Enna, Italy; antonino.maniaci@unikore.it; 3 Department of General Surgery, Faculty of Medicine, University of Mons (UMONS), Avenue du Champ de Mars, 6, B7000 Mons, Belgium; jerome.lechien@umons.ac.be; 4 Head and Neck Department, ENT & Oral Surgery Unit, G.B. Morgagni, L. Pierantoni Hospital, 47121 Forlì, Italy; giovanni.cammaroto@hotmail.com; 5 Department of Otorhinolaryngology-Head and Neck Surgery, Hospital Universitario Donostia, 20003 San Sebastian, Spain; chiesaestomba86@gmail.com; 6 Otolaryngology Head and Neck Unit, “Santo Spirito” Hospital, 65124 Pescara, Italy; pierre.guarino@asl.pe.it; 7 Department of Innovative Technologies in Medicine & Dentistry, University “G. d’Annunzio” Chieti-Pescara, 66013 Chieti, Italy; 8 Maxillofacial Surgery Unit, Department of Neurosciences, Reproductive and Odontostomatological Sciences, University of Naples Federico II, 80131 Naples, Italy; giovannisalzanomd@gmail.com; 9 Maxillofacial Surgery Operative Unit, Department of Medicine, Surgery and Pharmacy, University of Sassari, 07100 Sassari, Italy; lavaira@uniss.it; 10 ENT-HNS Department, Aix Marseille University, APHM, CNRS, IUSTI, La Conception University Hospital, 13284 Marseille, France; stephane.gargula@gmail.com; 11 Unit of Otolaryngology, Fondazione Policlinico Universitario Campus Bio-Medico, 00128 Rome, Italy; m.casale@unicampus.it (M.C.); a.moffa@unicampus.it (A.M.)

**Keywords:** Eustachian tube dysfunction, older adults, ageing, hearing loss, presbycusis, frailty, balloon dilation, narrative review

## Abstract

**Objective**: To critically summarize current evidence regarding the burden of Eustachian tube dysfunction (ETD) in older adults, focusing on age-related pathophysiology, diagnostic challenges, therapeutic strategies and its interaction with major geriatric clinical implications. **Methods**: This narrative review was conducted according to the Scale for the Assessment of Narrative Review Articles (SANRA). PubMed-indexed original studies, systematic reviews, meta-analyses, consensus statements and clinical guidelines addressing ETD and ageing were systematically identified and critically appraised. Evidence was synthesized narratively with particular emphasis on studies applicable to geriatric populations. **Results**: Current evidence demonstrates that ageing induces progressive structural and functional alterations throughout the Eustachian tube–middle ear unit, including cartilage calcification, muscular degeneration, mucosal atrophy and impaired neuromuscular coordination. These changes may increase susceptibility to ETD, which in turn can exacerbate presbycusis, vestibular dysfunction, communication disability and potentially contribute to frailty and cognitive vulnerability by increasing the cumulative burden of sensory impairment. However, evidence specifically derived from geriatric cohorts remains limited, particularly regarding long-term outcomes and therapeutic interventions and is insufficient to establish ETD as an independent determinant of these conditions. **Conclusions**: ETD should be recognized as an underdiagnosed and potentially modifiable contributor to hearing-related disability in older adults. Greater clinical awareness, standardized diagnostic pathways and prospective geriatric-focused studies are needed to clarify whether timely diagnosis and treatment of ETD can reduce the overall burden of hearing impairment, improve functional independence and enhance quality of life in ageing populations.

## 1. Introduction

Population ageing is profoundly reshaping the epidemiology of chronic sensory disorders, with older adults accounting for a disproportionate share of disability, loss of functional independence and healthcare utilization worldwide [1,2]. Within the Global Burden of Disease framework, hearing loss represents one of the leading causes of years lived with disability (YLDs), underscoring the substantial societal and healthcare impact of age-related auditory impairment [2]. In this context, Eustachian tube dysfunction (ETD) deserves greater clinical attention because even modest impairment of middle-ear ventilation, pressure regulation or conductive sound transmission may substantially amplify hearing-related disability in ears already affected by presbycusis [3,4,5,6]. Consequently, ETD has the potential to increase communication difficulties, listening effort, social isolation and healthcare needs, thereby contributing to the cumulative burden of sensory impairment in older adults [1,7,8].

Despite these potentially important clinical implications, ETD remains considerably under-recognized in geriatric medicine and has received relatively little attention in studies specifically addressing ageing populations [3,9]. This contrasts with the relatively extensive pediatric literature on ETD, particularly in relation to adenoid hypertrophy and middle-ear ventilation, where surgical management such as adenoidectomy has been specifically investigated [10].

Available epidemiological data indicate that ETD is not uncommon in later life; however, reported prevalence varies substantially according to the diagnostic approach employed, including administrative coding, symptom-based questionnaires, tympanometry or comprehensive clinical assessment [3,4,9,11,12,13].

In the United States, ETD prevalence was estimated at 4.6% overall in adults and 8.25% in those aged 65 years or older in a tympanometry-based national sample [14], while a SEER-Medicare analysis reported diagnosed ETD in 5.44% of elderly adults without upper aerodigestive tract malignancy [3].

This marked methodological heterogeneity, together with the frequent overlap between ETD, presbycusis, chronic middle-ear disease and other age-related otologic conditions, probably contributes to underdiagnosis and to an underestimation of its true clinical burden [4,9,11].

Beyond its direct otologic manifestations, ETD should no longer be regarded solely as a disorder of middle-ear ventilation. Rather, accumulating evidence suggests that it may represent a potentially modifiable contributor to hearing-related disability whose consequences extend to domains that are particularly relevant in geriatric medicine, including communication, balance, cognitive performance, frailty and quality of life [1,5,6,7,8]. Although direct evidence linking ETD to these outcomes remains limited, the biological plausibility of these interactions, together with their potential cumulative impact on health status and functional reserve, supports a broader interpretation of ETD within the spectrum of age-related vulnerability [3,9].

Against this background, this SANRA-based narrative review does not simply summarize the available literature but proposes a burden-oriented perspective for interpreting the role of ETD in older adults. By integrating current evidence from otology, audiology and geriatric medicine, the review examines how ETD may amplify the clinical consequences of presbycusis and other geriatric syndromes, discusses current diagnostic and therapeutic challenges and identifies priorities for future research. Ultimately, it aims to provide a conceptual framework for recognizing ETD as an underappreciated- and potentially modifiable-determinant of hearing-related disability and overall geriatric burden. The main epidemiological and clinical studies supporting the relevance of ETD in adult and older populations are summarized in Table 1.

## 2. Materials and Methods

This narrative review was designed to synthesize the current evidence regarding the burden, determinants, diagnosis and management of Eustachian tube dysfunction (ETD) in older adults using a structured, non-systematic methodology consistent with the Scale for the Assessment of Narrative Review Articles (SANRA) [16]. A narrative approach was considered more appropriate than a formal systematic review or meta-analysis because the available literature on ETD in ageing populations is highly heterogeneous, encompassing epidemiological studies, consensus statements, diagnostic-accuracy investigations, imaging studies and intervention reviews [3,9,17,18,19,20]. Moreover, older adults are rarely investigated as an independent study population, being more commonly included within broader adult cohorts or mixed-age populations [3,9]. Accordingly, a narrative synthesis was considered the most suitable methodology for critically integrating evidence derived from different study designs while preserving clinical applicability to geriatric otology. In accordance with SANRA recommendations, the review was structured around six methodological domains: justification of the review’s importance, explicit statement of objectives, transparent literature search, appropriate referencing, balanced evidence-based discussion and a reasoned endpoint focused on the clinical burden, diagnostic challenges and unmet needs of ETD in older adults [16].

A structured literature search was performed in PubMed/MEDLINE, Scopus, Web of Science, and Google Scholar from database inception to 10 May 2026. Search strategies combined controlled vocabulary and free-text terms related to ETD and ageing, including “Eustachian tube dysfunction”, “auditory tube dysfunction”, “Eustachian tube”, “older adults”, “elderly”, “geriatric”, “ageing”, “presbycusis”, “frailty”, “balance”, “falls”, “cognition”, “tympanometry”, “tubomanometry”, “sonotubometry”, “imaging”, “balloon dilation” and “balloon Eustachian tuboplasty”. Reference lists of consensus statements, diagnostic pathway studies, reviews on Eustachian tube function testing and landmark publications on medical and surgical management were manually screened to identify additional relevant articles not retrieved through the primary search. To ensure methodological consistency, only PubMed-indexed publications were included in the final reference list and all bibliographic data, including DOI information, were verified before manuscript preparation.

Eligible publications comprised PubMed-indexed original investigations, systematic reviews, meta-analyses, consensus statements, clinical practice recommendations and high-quality narrative reviews addressing ETD epidemiology, age-related pathophysiology, hearing impairment, vestibular dysfunction, cognition, frailty, diagnostic evaluation, imaging, and therapeutic interventions. Whenever available, studies specifically enrolling adults aged 65 years or older were prioritized. Because evidence exclusively derived from geriatric populations remains limited, studies including mixed adult cohorts with age-stratified analyses or findings considered clinically applicable to older adults were also included [3,9]. Titles and abstracts were initially screened for relevance, followed by full-text assessment whenever eligibility was uncertain. Publications focused exclusively on pediatric populations, conference abstracts without peer-reviewed full-text publication, duplicate reports, non-indexed literature and studies in which ETD could not be distinguished from other disorders of middle-ear ventilation according to contemporary consensus definitions were excluded [4].

The final evidence corpus included 48 PubMed-indexed publications and incorporated a broad range of study designs. Of these, 25 (52.1%) were published from 2021 onward, reflecting the emphasis placed on contemporary evidence; 25 were primary research articles, including 2 randomized controlled trials; 16 were review or evidence-synthesis articles and 7 consisted of consensus statements, guidelines, methodological contributions, clinical-guidance papers or public-health documents.

For each eligible study, data were extracted regarding country of origin, study design, sample size, age distribution, ETD subtype, diagnostic criteria, objective functional assessment, patient-reported outcome measures, therapeutic interventions and principal findings. Particular attention was devoted to the operational definition of ETD, since the Eustachian Tube Dysfunction Questionnaire-7 (ETDQ-7) represents a valuable instrument for quantifying symptom burden but should not be considered a stand-alone diagnostic tool [11,21]. Instead, current evidence supports a multimodal diagnostic approach integrating clinical history with otoscopy, tympanometry, tubomanometry, sonotubometry, endoscopic evaluation, functional testing and imaging when clinically indicated [9,11,17,18,21]. Beyond data extraction, individual studies were critically appraised with particular attention to methodological quality, diagnostic accuracy, risk of bias and applicability to geriatric clinical practice, thereby allowing interpretation of the available evidence within the broader context of hearing-related disability and ageing.

Because substantial heterogeneity was anticipated across study populations, ETD definitions, diagnostic methods and reported outcomes, quantitative synthesis was considered inappropriate. Instead, findings were narratively integrated according to the predefined thematic domains of the review, including epidemiology, age-related pathophysiological mechanisms, interactions with hearing loss, balance impairment, cognition and frailty, diagnostic challenges and current therapeutic strategies. Throughout the review process, particular emphasis was placed on understanding how ETD may contribute to the overall burden of hearing-related disability in older adults, rather than describing ETD as an isolated middle-ear disorder. The final manuscript was therefore developed to preserve full compliance with SANRA methodology through transparent reporting, balanced interpretation of the available evidence and a clinically oriented discussion highlighting current knowledge gaps and priorities for future research in geriatric otology [16].

## 3. Results

### 3.1. Pathophysiology and Age-Related Changes

Research question: What structural and functional changes occur in the Eustachian tube with advancing age and how do they contribute to dysfunction in older adults?

Ageing induces progressive structural and functional changes in the Eustachian tube (ET)–middle ear unit that provides the biological basis for chronic or recurrent ETD in later life. Histopathological studies have demonstrated calcification of the cartilaginous portion of the ET and fatty degeneration of the tensor veli palatini muscle, reducing both the elasticity of the tubal framework and the muscular force required for active opening during swallowing or yawning [22]. These anatomical findings are consistent with earlier physiological evidence showing reduced tubal efficiency in geriatric subjects and with functional observations that tubal opening becomes shorter and less effective with advancing age [23,24].

Mucosal ageing also contributes to ET dysfunction. Progressive epithelial atrophy, squamous metaplasia and impaired mucociliary clearance may reduce the ability of the ET to clear secretions and maintain stable middle-ear ventilation [25]. In older adults, these intrinsic changes often coexist with chronic rhinosinusitis, allergic rhinitis, laryngopharyngeal or gastroesophageal reflux and other inflammatory upper-airway conditions, which may further impair mucosal function and promote persistent pressure dysregulation [3,9]. Thus, ETD in the elderly rarely reflects a single mechanism; it more often results from the interaction between age-related tissue degeneration, chronic inflammation and reduced neuromuscular reserve.

The clinically relevant pathway can be summarized as follows: impaired active tubal opening and/or persistent middle-ear pressure dysregulation can reduce tympanic membrane–ossicular transmission and introduce a conductive component, particularly at lower frequencies; when superimposed on the typical high-frequency decline of presbycusis, this can broaden the effective auditory deficit, increase cognitive loading during communication and reduce hearing-rehabilitation efficiency [23,24,25].

These mechanisms are clinically important because they influence both diagnostic interpretation and treatment selection. Structural stiffening and muscular atrophy may limit the effectiveness of purely conservative measures based on active tubal opening, whereas inflammation-driven dysfunction may respond more favorably to treatment of rhinitis, chronic rhinosinusitis, reflux or obstructive sleep apnea when these conditions are present [9,26,27,28,29]. Similarly, a persistent conductive component caused by impaired middle-ear ventilation may have disproportionate functional consequences in patients with presbycusis, because even a modest additional air-bone gap can increase listening effort, reduce speech understanding and compromise hearing rehabilitation [5,6,8,30].

From a geriatric perspective, the key implication is that pathophysiology should not be described as an isolated anatomical process but translated into management decisions. Older adults with suspected ETD require evaluation of potentially reversible inflammatory drivers, objective assessment of middle-ear function and careful interpretation of symptoms within the broader context of presbycusis, frailty and multimorbidity [3,4,9,31,32]. Recognition of these age-related mechanisms therefore provides the rationale for an individualized treatment strategy, in which the goal is not only restoration of middle-ear pressure regulation but also reduction in hearing-related disability and improvement in functional outcomes.

### 3.2. ETD and Presbycusis

Research question: How does Eustachian tube dysfunction interact with age-related sensorineural hearing loss (presbycusis) in older adults?

Presbycusis is the most prevalent cause of hearing impairment in older adults and primarily reflects progressive degeneration of cochlear sensory cells, auditory neurons and central auditory pathways [1,2,30]. However, age-related sensorineural hearing loss frequently coexists with Eustachian tube dysfunction (ETD), introducing a conductive component that may substantially amplify hearing-related disability beyond that expected from cochlear degeneration alone [5,6]. Even modest abnormalities in middle-ear ventilation or pressure regulation can reduce sound transmission efficiency, increase air-bone gaps and contribute to fluctuating hearing thresholds, thereby complicating audiological assessment and hearing rehabilitation [5,6,9,17].

The frequency characteristics of the two components are clinically useful. Age-related sensorineural hearing loss typically produces a high-frequency sloping audiometric pattern, with progressively greater threshold deterioration at higher frequencies as age advances [5,6]. By contrast, experimental and clinical data on negative middle-ear pressure show more prominent threshold effects at lower frequencies, including low-frequency air-bone gaps [5,6]. Thus, ETD superimposed on presbycusis may produce a mixed pattern in which a potentially reversible low-frequency conductive component coexists with an irreversible high-frequency sensorineural deficit.

Prospective clinical studies support this interaction. In a Brazilian cohort of 103 adults aged ≥ 60 years, presbycusis was identified in 59.2% of participants, whereas abnormal tympanograms and absent acoustic reflexes were observed in 39.3% and 37.9%, respectively. Importantly, both abnormalities were significantly more frequent among individuals with moderate-to-severe age-related hearing loss, suggesting that subclinical middle-ear dysfunction commonly accompanies presbycusis and may contribute to unexpectedly poor auditory performance [5]. Similar findings were reported in a cross-sectional African study involving 121 adults aged 61–96 years, in which silent middle-ear malfunction (S-MEM)—defined as abnormal tympanometric findings despite a normal otoscopic examination—was identified in 21.5% of participants. Although these patients frequently lacked overt conductive symptoms, audiometric testing demonstrated conductive or mixed hearing components capable of aggravating pre-existing sensorineural hearing loss [6].

Beyond its audiometric implications, ETD may also influence hearing rehabilitation outcomes. Conductive dysfunction superimposed on presbycusis can reduce speech discrimination, particularly in noisy environments, increase listening effort and limit the perceived benefit of hearing aids despite appropriate device fitting [5,6,7,8,30]. Consequently, unexplained variability in hearing thresholds or reduced hearing-aid satisfaction should prompt careful evaluation of middle-ear function rather than being attributed exclusively to cochlear degeneration [9,17]. This concept is particularly relevant in geriatric populations, where multiple coexisting auditory and systemic conditions frequently interact to influence communication abilities [7,8].

Interestingly, Sogebi and colleagues also identified previous head injury, diabetes mellitus, osteoarthritis and absent acoustic reflexes as independent predictors of silent middle-ear malfunction, emphasizing that ETD in older adults should be interpreted within the broader context of multimorbidity rather than as an isolated otologic disorder [6]. Because many of these conditions become increasingly prevalent with advancing age, their coexistence may further increase susceptibility to chronic ET dysfunction and contribute to the overall complexity of hearing impairment in geriatric patients [6,8].

Comprehensive audiological evaluation—including tympanometry and objective assessment of middle-ear function—should therefore be considered an integral component of hearing assessment in older adults, particularly before hearing-aid prescription or cochlear implantation [9,17]. Recognizing and treating reversible conductive components may represent one of the few potentially modifiable opportunities to reduce hearing-related disability in ageing populations [1,5,6,7].

### 3.3. ETD, Balance Disorders and Falls

Research question: How does Eustachian tube dysfunction contribute to balance disorders and increase the risk of falls in older adults?

Balance impairment and falls represent major contributors to morbidity, loss of independence, institutionalization and healthcare utilization in older adults [33,34,35]. Although the relationship between Eustachian tube dysfunction (ETD) and falls has received relatively limited scientific attention, current physiological and clinical evidence supports a biologically plausible association between impaired middle-ear pressure regulation and vestibular dysfunction [4,33,36].

When the Eustachian tube fails to open or close appropriately, persistent negative middle-ear pressure may alter the mechanical forces transmitted through the ossicular chain, resulting in abnormal displacement of the stapes footplate and distortion of pressure gradients across the oval and round windows. These alterations may disrupt inner-ear fluid homeostasis and stimulate vestibular sensory organs, producing alternobaric vertigo. Alternobaric vertigo is a pressure-induced vestibular syndrome caused by unequal middle-ear pressure between the two ears, usually during or after changes in ambient pressure when pressure equalization is asymmetric. It may present with transient vertigo, nausea, nystagmus and postural instability and is best documented in baro-challenge settings [36]. In unilateral ETD, patients may deviate toward the affected side during ambulation, whereas bilateral dysfunction more frequently manifests as oscillopsia, unsteadiness and impaired postural control rather than true rotational vertigo [36]. Although central vestibular compensation often reduces symptoms over time, chronic or recurrent ETD may result in persistent disequilibrium, particularly in individuals with reduced physiological reserve [33,36].

The epidemiological burden of vestibular dysfunction further supports the potential clinical importance of ETD. Nationally representative data from the U.S. National Health and Nutrition Examination Survey demonstrated that 35.4% of adults aged 40 years or older had objective vestibular dysfunction, with prevalence increasing steeply with age [34]. Vestibular dysfunction was also strongly associated with dizziness and falls and participants with symptomatic vestibular dysfunction had markedly increased odds of falling [34]. Although these studies did not specifically investigate ETD, they provide a strong rationale for considering ETD as one of several potentially reversible factors capable of aggravating vestibular instability in susceptible individuals [33,34,35,36].

Importantly, ETD rarely occurs in isolation in older adults. Visual impairment, peripheral neuropathy, sarcopenia, osteoarthritis, polypharmacy and cognitive impairment frequently coexist, each contributing to impaired postural control [34,35]. Within this multifactorial framework, ETD may act as an additional stressor capable of reducing balance reserve and increasing vulnerability to falls. While its isolated contribution is likely modest, its cumulative effect in multimorbid older adults may be clinically significant [33,34,35].

From a clinical perspective, the relevance of ETD extends beyond the occurrence of transient vertigo. Even subtle vestibular symptoms may reduce confidence during walking, increase fear of falling, limit physical activity, accelerate functional decline and ultimately contribute to frailty and loss of independence [33,34,35]. Consequently, older adults presenting with dizziness, unexplained imbalance or recurrent falls should undergo careful otologic and middle-ear evaluation, particularly when symptoms fluctuate with pressure changes or coexist with hearing complaints [4,9,35,36]. Although direct evidence linking ETD treatment to fall prevention remains unavailable, early recognition and appropriate management of ETD may represent a potentially modifiable strategy for reducing the overall burden of balance-related disability in ageing populations [33,34,35,36].

### 3.4. ETD and Cognitive Decline

Research question: Does chronic Eustachian tube dysfunction contribute to cognitive decline in older adults?

Cognitive decline is increasingly recognized as a multifactorial process in which sensory impairment represents an important and potentially modifiable risk factor [7,30,37,38]. Although no study has directly identified Eustachian tube dysfunction (ETD) as an independent determinant of cognitive decline or dementia, several lines of evidence suggest that chronic middle-ear dysfunction may indirectly contribute to cognitive vulnerability by exacerbating hearing impairment, increasing cognitive loading and reducing the quality and consistency of auditory input [5,6,30,37,38]. Within this framework, ETD may represent one component of the broader pathway linking hearing loss to adverse cognitive outcomes in ageing populations [7,30,37,38].

The relationship between hearing impairment and cognition has been extensively investigated [7,30,38]. Chronic ETD may exacerbate presbycusis by adding a conductive component that reduces auditory input, thereby increasing cognitive load during communication and potentially contributing to social withdrawal and cognitive vulnerability in older adults [5,6,7,30,37,38].

Clinical evidence supporting this hypothesis is emerging. In older adults with age-related hearing loss and chronic otitis media, Gao and colleagues reported improvement in conductive hearing, Montreal Cognitive Assessment scores and quality of life after middle-ear surgery [37]. This finding supports the concept that at least part of hearing-related cognitive impairment may be partially reversible when conductive deficits are corrected.

More broadly, longitudinal studies show that hearing loss is associated with faster cognitive decline [30] and the 2024 Lancet Standing Commission identifies untreated hearing loss as a major potentially modifiable dementia risk factor [7]. Although this evidence primarily concerns hearing loss as a whole rather than ETD specifically, it provides a compelling conceptual framework through which the contribution of chronic conductive hearing impairment may be interpreted [7,30,38].

ETD should therefore not be regarded as an established cause of cognitive impairment but rather as a potential amplifier of hearing-related cognitive burden, particularly when persistent conductive dysfunction coexists with presbycusis [5,6,30,37,38].

The clinical importance of this hypothesis lies in recognizing ETD as one of the few potentially modifiable contributors to hearing-related disability [1,7,8,30,38]. By reducing additional conductive hearing loss, optimizing auditory rehabilitation and improving communication, appropriate management of ETD may help decrease listening effort, facilitate social participation and ultimately reduce the cumulative burden of sensory impairment that has been associated with cognitive vulnerability in older adults [1,7,8,30,37,38].

### 3.5. ETD in Frail and Multimorbid Older Adults

Research question: Why is Eustachian tube dysfunction particularly relevant in frail and multimorbid older adults?

Frailty and multimorbidity substantially modify the clinical expression and overall burden of Eustachian tube dysfunction (ETD) in older adults [31,32]. Rather than representing an isolated otologic disorder, ETD frequently develops within a complex biological environment characterized by chronic inflammation, immunosenescence, sarcopenia, impaired neuromuscular coordination and reduced physiological reserve [22,23,31,32].

Older adults commonly present with multiple chronic conditions, including chronic rhinosinusitis, allergic rhinitis, gastroesophageal reflux disease, diabetes mellitus, chronic obstructive pulmonary disease and cardiovascular disease, that may contribute to persistent nasopharyngeal inflammation, impaired mucosal function and altered Eustachian tube physiology [4,9,27,39]. Obstructive sleep apnea may represent an additional adult comorbidity relevant to ET physiology, as ETS-7-based observational data and recent systematic evidence suggest an association between sleep-disordered breathing, impaired tubal opening, and altered middle-ear pressure regulation [26,27].

Importantly, ETD in older adults should not be equated with obstructive or dilatory ETD. Although age-related muscular degeneration and structural stiffening may impair active opening, weight loss, chronic illness, nasopharyngeal or muscular atrophy and neuromuscular disorders can also predispose to patulous ETD [4,40]. Patulous ETD is characterized by excessive tubal patency and typically presents with autophony or breathing-related aural symptoms; its diagnostic and therapeutic approach differs fundamentally from obstructive ETD. Accurate phenotyping is therefore essential before attributing symptoms to obstruction or considering BDET.

The clinical presentation of ETD may be nonspecific in frail patients because fluctuating hearing, poor hearing-aid benefit, imbalance, sleep disturbance and communication difficulties overlap with other age-related conditions [3,8,9,31,32]. This overlap increases the risk of both underdiagnosis and misclassification. Symptoms such as autophony should prompt consideration of patulous rather than obstructive dysfunction, whereas persistent negative middle-ear pressure, effusion or retraction support a dilatory/obstructive phenotype [4,40,41].

ETD may indirectly contribute to social isolation, depressive symptoms, reduced participation in daily activities and increasing dependence during interpersonal communication [1,7,8,30]. Although these outcomes cannot be attributed exclusively to ETD, they are highly consistent with the multidimensional pathways through which sensory impairment contributes to frailty progression and cognitive vulnerability in later life [7,8,30,31,32,38].

Management of ETD in frail older adults is further complicated by multimorbidity and polypharmacy [31,32]. Anticholinergic medications, sedatives, antihistamines, psychotropic drugs and other commonly prescribed treatments may influence mucosal hydration, vestibular compensation, alertness or cognitive performance, thereby masking ETD-related symptoms or complicating their interpretation [31,32,33,34,35]. Similarly, reduced mobility, cognitive impairment and limited access to specialist assessment may delay diagnosis and reduce adherence to both medical treatment and hearing rehabilitation [1,7,8,31,32].

Despite these important clinical considerations, frail older adults remain markedly underrepresented in studies evaluating both medical treatment and balloon Eustachian tuboplasty [9,19,20]. Consequently, most current diagnostic algorithms and therapeutic recommendations are derived from younger adult populations and may not adequately reflect the biological complexity or multidimensional needs of geriatric patients [4,9,11,19,20,31,32].

From a geriatric perspective, ETD should therefore be considered within a comprehensive geriatric framework in order to facilitate earlier diagnosis, optimize hearing rehabilitation, improve communication and potentially reduce functional decline [1,7,8,30,31,32].

A summary of the clinical implications in older adults driven by ETD, along with their certainty grade, discussed in the previous paragraphs, is reported in Table 2.

### 3.6. Diagnostic Challenges in Older Adults

Research question: Why is Eustachian tube dysfunction frequently underdiagnosed in older adults?

The diagnosis of Eustachian tube dysfunction (ETD) in older adults remains particularly challenging because age-related physiological changes, multimorbidity and overlapping otologic disorders frequently obscure its clinical presentation [3,4,9,31,32]. Unlike younger individuals, older patients often present with nonspecific or fluctuating symptoms that may be mistakenly attributed to presbycusis, chronic otitis media, vestibular disease or simply to physiological ageing [3,8,9,30,33,37]. Consequently, ETD is likely to be substantially underdiagnosed in geriatric practice, contributing to persistent hearing-related disability and delayed therapeutic intervention [3,9].

A major limitation of current diagnostic pathways is the absence of a single reference standard for ETD [4,9,11]. The international consensus statement defines ETD as a syndrome characterized by symptoms and signs of pressure dysregulation of the middle ear, emphasizing that diagnosis should integrate clinical history with objective evidence of impaired Eustachian tube function rather than relying on symptoms alone [4]. Accordingly, contemporary assessment requires a multimodal approach in which no individual investigation is considered sufficient to establish or exclude the diagnosis [9,11,17,18,21].

The principal diagnostic tools currently used for ETD, together with their strengths, limitations and applicability in older adults, are summarized in Table 3.

From a geriatric perspective, diagnostic interpretation should extend beyond isolated otologic findings [8,31,32]. Comprehensive assessment should include hearing thresholds, speech discrimination, hearing-aid performance, balance symptoms, cognitive status, medication review and major comorbidities, recognizing that ETD frequently interacts with multiple age-related conditions rather than occurring as an isolated disorder [1,7,8,30,31,32,33,34,35]. This multidimensional approach is particularly important in frail older adults, in whom relatively mild conductive dysfunction may disproportionately affect communication, rehabilitation outcomes and functional independence [1,5,6,7,8,31,32].

Failure to recognize ETD in older adults may lead to persistent conductive hearing impairment, unnecessary escalation of hearing-aid amplification, delayed rehabilitation, repeated healthcare consultations and reduced quality of life [1,3,5,6,8,9]. Since ETD represents one of the few potentially reversible contributors to hearing-related disability, early identification through a structured, multimodal diagnostic pathway may substantially reduce the cumulative burden of sensory impairment in ageing populations [1,4,7,8,9,11].

### 3.7. Therapeutic Management and Future Perspectives

Research question: How should Eustachian tube dysfunction be managed in older adults?

The therapeutic management of ETD in older adults should be reframed around clinical benefit rather than anatomical correction alone. In younger adults, treatment is often directed primarily toward aural fullness, pressure symptoms or normalization of tympanometry. In older patients, however, the therapeutic target is broader: improvement of communication, stabilization of hearing rehabilitation, reduction in listening effort and preservation of functional independence [1,7,8,30]. Management should be individualized according to ETD subtype, symptom severity, objective evidence of middle-ear pressure dysregulation, reversibility of associated upper-airway disease and patient-specific vulnerability [3,9,31,32,35].

A practical first step is to distinguish obstructive or dilatory ETD from patulous ETD and from non-ETD mimics such as temporomandibular disorders, chronic otitis media without active tubal dysfunction, Meniere-like syndromes or nonspecific auditory complaints. This distinction is essential because treatments that may benefit obstructive ETD, including decongestant-like strategies or balloon dilation, may be inappropriate or potentially harmful in patulous dysfunction [4,9,11,40,41]. In older adults, diagnostic confirmation should therefore precede escalation of treatment, particularly before invasive procedures are considered.

The main therapeutic options for ETD in older adults, including conservative, medical, surgical and geriatric-informed strategies are summarized in Table 4.

#### 3.7.1. Conservative Management

Conservative management remains the initial approach for many patients, especially when symptoms are mild, intermittent or associated with reversible inflammatory disease. Patient education, gentle swallowing or yawning exercises, avoidance of rapid pressure changes when feasible and cautious auto-inflation may provide symptomatic benefit in selected individuals [19,20]. However, these strategies may be less effective in older adults with muscular atrophy or reduced neuromuscular reserve, and pressure-based maneuvers should be recommended carefully, since rare inner-ear complications after ET insufflation have been reported [20,22,23].

The evidence supporting pharmacological treatment for chronic adult ETD remains limited. A recent systematic review and meta-analysis of medical management in adults found no level 1 evidence supporting routine pharmacological therapy and reported that intranasal corticosteroids were not effective for chronic symptoms in most available studies [19]. These findings are consistent with earlier intervention reviews showing uncertainty regarding the efficacy of decongestants, antihistamines, systemic corticosteroids and non-surgical pressure-equalization strategies for persistent adult ETD [19,20]. In geriatric patients, the threshold for routine or prolonged medical therapy should be even higher because polypharmacy, anticholinergic burden, sedation, hypertension, diabetes and osteoporosis may increase treatment-related risks [31,32]. Medical therapy should therefore be directed toward clearly identifiable comorbid conditions rather than prescribed as an undifferentiated treatment for chronic ETD.

#### 3.7.2. Treatment of Associated Upper-Airway Disease

Management of associated nasal, nasopharyngeal and upper-airway disease is particularly relevant in older adults. Chronic rhinosinusitis, allergic rhinitis, laryngopharyngeal or gastroesophageal reflux and persistent nasopharyngeal inflammation may contribute to ET mucosal edema and impaired tubal opening [3,9,27,28,29,39]. In patients with active inflammatory disease, targeted therapy such as saline irrigation, intranasal corticosteroids, antihistamines, anti-reflux treatment or treatment of chronic rhinosinusitis may be clinically reasonable, although the expected benefit should be interpreted as treatment of a contributing condition rather than direct evidence-based therapy for primary structural ETD [9,19,20].

Recent evidence also suggests that nasal obstruction and sleep-disordered breathing deserve attention within a multimodal treatment model. Surgical correction of non-sinusitis-related nasal obstruction has been associated with significant improvement in ETDQ-7 scores over postoperative follow-up, supporting the concept that nasal airflow and nasopharyngeal mechanics may influence ETD symptoms in selected adults [27,28,29,44]. Similarly, observational data and systematic evidence indicate that obstructive sleep apnea and continuous positive airway pressure use may be associated with middle-ear pressure changes, impaired tubal opening or ET-related symptoms, although causality and optimal management remain uncertain [26,27]. In older adults with multimorbidity, identifying and treating these conditions may improve global airway health and may reduce one component of the ETD burden.

#### 3.7.3. Ventilation Tubes

Tympanostomy or ventilation tubes remain an effective option for selected adults with persistent middle-ear effusion, chronic negative middle-ear pressure or symptomatic conductive hearing loss refractory to conservative treatment [19,20]. Their main advantage is immediate pressure equalization and often rapid improvement in aural fullness or conductive hearing. This may be particularly useful in older adults in whom even a small conductive component worsens presbycusis and interferes with hearing-aid fitting [5,6,30].

However, ventilation tubes should be presented as a bypass procedure rather than a treatment that restores physiological ET function [4,19,20]. Recurrent insertion, chronic otorrhea, persistent tympanic membrane perforation and chronic middle-ear changes remain relevant limitations, especially in frail patients or in those with chronic inflammatory disease, diabetes or impaired tissue healing [31,32]. Therefore, ventilation tubes may be appropriate when the therapeutic goal is rapid control of middle-ear pressure or hearing rehabilitation, but they do not eliminate the need to evaluate the underlying cause of ETD.

#### 3.7.4. Balloon Eustachian Tuboplasty

Balloon Eustachian tuboplasty or balloon dilation of the Eustachian tube (BDET/BET) has become the most important procedural development for chronic obstructive ETD. The procedure aims to dilate the cartilaginous portion of the ET, reduce mucosal inflammation and remodeling, and improve the ability of the tube to open during physiological maneuvers [20,41,45,46]. Consensus recommendations support consideration of BDET in adults with chronic obstructive ETD when symptoms persist despite appropriate medical management and when objective evidence of tubal dysfunction is present [41].

The evidence base has expanded in recent years but remains heterogeneous. Earlier systematic reviews and meta-analyses reported improvements in ETDQ-7 scores, tympanometric findings, Valsalva ability and patient-reported outcomes after BDET, although study design, selection criteria and follow-up duration varied substantially [20,45,46]. A randomized controlled trial with six-month follow-up suggested that BDET may improve tympanometric and otoscopic outcomes in a selected group of adults with mild chronic ETD, although differences in mean ETDQ-7 scores were not significant [47]. A 2024 systematic review including studies published through March 2024 concluded that BDET may reduce symptom burden with a low rate of mostly self-limited complications, but also emphasized heterogeneity and risk of bias across the available literature [45].

More recent evidence further supports a cautiously favorable interpretation. A meta-analysis of randomized controlled trials published in 2026 reported that BDET increased the likelihood of achieving a type A tympanogram in adults with chronic ET dysfunction and improved air-bone gap closure when performed with tympanoplasty in selected middle-ear disease settings [46]. A multicenter randomized controlled trial in adults with chronic suppurative otitis media and dilatory ETD found that BET combined with medical management was superior to medical management alone for normalization of the Valsalva maneuver, ETDQ-7 improvement and short-term air-bone gap reduction, without serious device- or procedure-related adverse events during early follow-up [48].

Nevertheless, several limitations remain highly relevant to older adults. Most BDET studies include mixed adult cohorts with limited age-stratified data and relatively few frail patients older than 70 years [20,45,46,48]. Older adults may have a different balance between inflammatory, muscular and structural mechanisms of ETD; cartilage calcification and muscular degeneration could theoretically reduce response in some patients, whereas correction of even modest conductive dysfunction may produce meaningful functional improvement in others [22,23]. Therefore, BDET should not be presented as a universal treatment for geriatric ETD but as one option within a phenotype-driven and function-oriented pathway.

#### 3.7.5. Patient Selection, Expected Benefit and Safety

In older adults, treatment selection should integrate otologic phenotype with geriatric priorities. Patients with intermittent symptoms and reversible inflammatory triggers may benefit most from conservative and upper-airway-directed therapy. Patients with persistent effusion or negative middle-ear pressure and clinically relevant conductive loss may benefit from ventilation tubes, particularly when rapid hearing optimization is needed. Patients with chronic obstructive ETD, persistent symptoms despite optimized medical treatment and objective evidence of impaired tubal function may be considered for BDET, provided that patulous ETD, nasopharyngeal lesions and alternative diagnoses have been excluded [4,9,19,20,41].

In many geriatric patients, a modest improvement in hearing-aid tolerance or communication may be more clinically meaningful than complete normalization of tympanometry. A patient-centered perspective should guide both procedural and non-procedural management.

#### 3.7.6. Future Therapeutic Perspectives

Recent umbrella-review evidence confirms that interventions for obstructive ETD are promising but still limited by heterogeneous indications, variable outcome measures and incomplete long-term safety data [20]. Future studies should therefore move beyond purely otologic endpoints and include outcomes that matter to older adults: hearing-aid benefit, speech-in-noise performance, communication ability, quality of life, falls, cognitive status, frailty trajectory, healthcare utilization and functional independence [1,7,8,31,32,35]. Trials should also report age-stratified outcomes and include frail or multimorbid patients, who are currently underrepresented despite being those most likely to experience disproportionate functional consequences from untreated ETD.

The most clinically useful future model is likely to be multidisciplinary. Otolaryngologists, audiologists, geriatricians, sleep physicians, rhinologists and primary-care clinicians should collaborate to identify reversible contributors to ETD, optimize hearing rehabilitation and select invasive treatments only when the expected functional benefit justifies procedural risk [3,8,31,32]. In this framework, successful ETD management should be understood not simply as correction of middle-ear pressure but as an opportunity to reduce one potentially modifiable component of hearing-related disability in ageing populations.

## 4. Discussion

The present narrative review highlights that Eustachian tube dysfunction (ETD) should no longer be regarded exclusively as a disorder of middle-ear ventilation but rather as an underrecognized contributor to hearing-related disability in older adults [1,3,4,7,8]. By integrating evidence from otology, audiology and geriatric medicine, the available literature suggests that ETD may substantially amplify the clinical consequences of age-related sensory decline through complex interactions with presbycusis, vestibular dysfunction, frailty and multimorbidity [5,6,7,8,30,31,32,33,34,35,37,38]. Although direct geriatric evidence remains limited, the cumulative data consistently indicate that even relatively mild impairment of Eustachian tube function may have disproportionately important clinical consequences in ageing populations [3,9,31,32].

One of the principal findings emerging from this review is that ageing affects virtually every anatomical and functional component of the Eustachian tube. Progressive cartilage calcification, degeneration of the tensor and levator veli palatini muscles, connective-tissue remodeling, epithelial atrophy and impaired mucociliary clearance collectively reduce the efficiency of middle-ear pressure regulation [22,23,24,25]. Unlike transient ETD occurring during childhood or acute upper respiratory infections, ETD in older adults develops within a biological environment characterized by reduced physiological reserve, chronic inflammation, immunosenescence and multisystem degeneration [31,32]. Consequently, identical degrees of tubal dysfunction may produce substantially greater functional impairment in older individuals than in younger adults [1,8,31,32].

A second important observation concerns the interaction between ETD and presbycusis. Sensorineural hearing loss remains the dominant cause of age-related auditory impairment; however, superimposed conductive dysfunction may further reduce speech perception, increase listening effort and impair hearing rehabilitation [5,6,30]. The available evidence suggests that abnormal middle-ear function is relatively common among older adults with presbycusis, indicating that part of the hearing disability traditionally attributed exclusively to cochlear degeneration may instead reflect a combination of sensorineural and conductive mechanisms [5,6]. This distinction is clinically relevant because, unlike cochlear ageing, conductive dysfunction associated with ETD may be at least partially reversible [5,6,19,20,45,46].

The present review also considers the possible relationship between ETD and balance symptoms; however, this association should be interpreted cautiously, as direct evidence linking ETD to chronic vertigo in older adults is limited, while age-related vestibular decline and common geriatric comorbidities may independently account for dizziness and imbalance [33,34,35,36]. Importantly, ETD should not be considered an isolated cause of falls but rather one of several potentially modifiable contributors acting within the multifactorial model of geriatric instability [31,32,35]. Future longitudinal studies should determine whether diagnosis and treatment of ETD can reduce balance-related disability or improve rehabilitation outcomes following falls [20,35,41,46].

The relationship between ETD and cognitive decline deserves equally cautious interpretation. No study has yet demonstrated that ETD independently increases the risk of dementia. Nevertheless, current evidence strongly supports hearing loss as one of the major modifiable risk factors for cognitive decline in later life [7,30,38]. Within this framework, chronic ETD may reasonably be viewed as a condition capable of amplifying hearing-related cognitive burden by reducing auditory input, increasing communication effort and promoting social withdrawal [7,8,30,37,38]. This conceptual model is biologically plausible and consistent with contemporary theories linking sensory deprivation to cognitive vulnerability, although direct confirmation through prospective geriatric studies remains necessary [7,37,38].

Perhaps the most original aspect emerging from this review is the interaction between ETD, frailty and multimorbidity. Frail older adults frequently present with overlapping chronic diseases, sarcopenia, polypharmacy, vestibular impairment and reduced adaptive capacity [31,32,35]. Under these circumstances, ETD rarely represents an isolated otologic condition but instead becomes one component of a broader syndrome of sensory and functional vulnerability [8,31,32]. Consequently, its clinical impact should be interpreted within the framework of Comprehensive Geriatric Assessment (CGA), recognizing that relatively small impairments in hearing or balance may translate into substantial reductions in autonomy, participation and quality of life [1,8,31,32].

Another important finding concerns the persistent diagnostic uncertainty surrounding ETD. Despite considerable advances in tubomanometry, sonotubometry, endoscopic assessment and imaging, no universally accepted diagnostic reference standard currently exists [4,9,11,12,17,18,21,39]. This limitation is particularly relevant in older adults because symptoms are frequently atypical, fluctuating or masked by presbycusis and multimorbidity [3,5,6,9,31,32] A multidimensional diagnostic strategy integrating symptom assessment, objective functional testing, audiological evaluation and geriatric assessment therefore appears more appropriate than reliance on any single investigation [9,11,17,18,21,32]. Development of standardized diagnostic pathways specifically validated in geriatric populations should represent a research priority [9,31,32].

Therapeutic management presents similar challenges. Conservative measures, treatment of associated inflammatory disorders, ventilation tubes and balloon Eustachian tuboplasty all have roles in appropriately selected patients [12,19,20,41,45,46]. However, almost all available evidence derives from younger adult populations, whereas frail older adults remain markedly underrepresented in clinical trials [9,20,45,46]. Future intervention studies should therefore evaluate outcomes that extend beyond symptom scores or tympanometric normalization to include hearing rehabilitation, communication ability, quality of life, falls, cognitive function, frailty status and maintenance of functional independence [1,7,8,20,31,32,35,41,46].

The findings of this review should be interpreted in light of several limitations. First, the review is narrative in design and therefore does not provide quantitative estimates of effect size [16]. Second, the literature specifically addressing adults aged 65 years and older remains remarkably limited, requiring cautious extrapolation from mixed adult cohorts [3,9,20,45,46]. Third, considerable heterogeneity exists regarding diagnostic criteria, outcome measures and ETD phenotyping across published studies, limiting direct comparison between investigations [4,9,11,17,18,21]. Finally, many proposed interactions between ETD, frailty, falls and cognition remain biologically plausible rather than definitively demonstrated, highlighting the need for prospective geriatric-specific research [7,9,32,35,38]. An additional limitation is the variability in ETD phenotyping, particularly regarding the distinction between obstructive/dilatory and patulous ETD, which represent pathophysiologically distinct entities and may complicate the comparison and interpretation of findings across studies.

Future investigations should prioritize prospective longitudinal studies specifically enrolling older adults with well-defined ETD. Standardized diagnostic criteria, objective functional assessment and validated patient-reported outcome measures should be combined with geriatric endpoints including hearing-related quality of life, communication ability, frailty, falls, cognitive performance and maintenance of functional independence [1,7,8,9,31,32,35]. Randomized controlled trials evaluating balloon Eustachian tuboplasty and other therapeutic interventions should include frail older adults and report outcomes that extend beyond middle-ear physiology [20,41,45,46]. Such studies will be essential to determine whether timely recognition and treatment of ETD can meaningfully reduce the burden of hearing-related disability in ageing populations [1,7,8,31,32].

## 5. Conclusions

Eustachian tube dysfunction (ETD) should no longer be regarded exclusively as a disorder of middle-ear ventilation [4]. Consistent with its primary aim, this review provides a conceptual framework for interpreting ETD in older adults as a potentially modifiable contributor to hearing-related disability, integrating age-related changes in Eustachian tube function, middle-ear pressure dysregulation, interaction with presbycusis, diagnostic phenotyping and individualized management within the broader context of geriatric vulnerability.

In older adults, progressive structural degeneration of the Eustachian tube, together with multimorbidity, frailty and age-related sensory decline, creates a unique clinical context in which even modest impairment of tubal function may substantially amplify hearing-related disability [1,3,8,22,23,24,25,30,31,32]. Although direct evidence specifically addressing geriatric populations remains limited, the available literature consistently supports the concept that ETD may contribute to the cumulative burden of communication difficulties, reduced hearing rehabilitation, balance impairment and functional decline [1,5,6,7,8,31,32,33,34,35]. However, current evidence is insufficient to establish ETD as an independent determinant of falls, frailty or cognitive decline.

The findings synthesized in this review suggest that ETD represents one of the few potentially modifiable contributors to hearing-related disability in ageing populations [1,3,7,8]. Early recognition through comprehensive audiological and otologic assessment, together with individualized management addressing both Eustachian tube dysfunction and associated comorbidities, may improve communication, optimize hearing rehabilitation and enhance quality of life in selected older adults [9,11,17,20,21,41,45,46].

Multidisciplinary collaboration among otolaryngologists, geriatricians, audiologists and neurologists is essential to address the complex interplay between ETD, presbycusis, balance disorders and cognitive impairment.

Future research should move beyond traditional otologic outcome measures and determine whether diagnosis and treatment of ETD can influence clinically meaningful geriatric outcomes, including falls, frailty, cognitive trajectories, functional independence and healthcare utilization [1,7,8,31,32,35]. Such evidence will be essential to establish the true clinical burden of ETD and to define its role within contemporary models of healthy ageing and comprehensive geriatric care [1,7,8,31,32].

## Figures and Tables

**Table 1:** Key epidemiological and clinical evidence on ETD in adults and older adults.

Study	Design/Population	ETD Assessment	Main Findings	Relevance to Older Adults
Browning & Gatehouse, 1992 [13]	Population-based adult study, UK	Otoscopic and audiological criteria	Presumptive adult ETD prevalence approximately 0.9%	Landmark adult prevalence estimate; shows ETD is not exclusively pediatric
Shan et al., 2019 [14]	Population-based analysis, U.S. adults	Population-based estimate	Estimated prevalence of ETD in U.S. adults approximately 4.6%	Supports a substantial adult burden
McCoul et al., 2019 [15]	Claims-based adult utilization study	Administrative coding/utilization patterns	More than 1.2 million adults diagnosed; approximately 11% chronic ETD	Demonstrates health-care burden and chronic disease relevance
Fischer et al., 2020 [3]	Retrospective cohort, U.S. patients > 65 years	Diagnostic coding/clinical review	ETD prevalence 5.44% in older adults without malignancy and 9.08% with malignancy	Core epidemiological study directly focused on the elderly population
Sogebi et al., 2015 [5]	Prospective study, adults ≥ 60 years	Audiometry, tympanometry, acoustic reflexes	Abnormal tympanograms and absent reflexes were common in older adults with presbycusis	Highlights coexistence of middle-ear dysfunction and age-related hearing loss
Sogebi et al., 2017 [6]	Cross-sectional elderly cohort, adults 61–96 years	Impedance/tympanometric evaluation	Silent middle-ear malfunction identified in 21.5% of participants	Supports the relevance of subclinical middle-ear dysfunction in hearing rehabilitation

Key epidemiological and clinical studies on ETD in adults and older adults are summarized in Table 1.

**Table 2:** Summary of evidence, certainty and clinical implications in older adults.

Domain	What Current Evidence Supports	Certainty	Clinical Implication
Age-related ET changes [22,23,24,25]	Structural and functional ageing changes are documented	Established biological evidence	May alter ET opening/clearance; does not by itself define ETD phenotype
ETD + presbycusis [5,6]	Middle-ear abnormalities can coexist with age-related SNHL and add a conductive component	Moderate/direct for auditory interaction	Evaluate reversible conductive loss before attributing all disability to presbycusis
Pressure-related vertigo [36]	Asymmetric pressure equalization can cause alternobaric vertigo	Direct in pressure-challenge contexts	Consider ET assessment when dizziness is pressure-triggered
ETD + falls [33,34,35]	Older adults commonly have vestibular dysfunction/falls, but ETD-specific fall data are lacking	Hypothesis-generating	Do not attribute recurrent falls to ETD without pressure-related/otologic evidence
ETD + cognition [7,30,37,38]	Hearing loss is associated with cognition; direct ETD-cognition evidence is absent	Hypothesis-generating	Avoid causal claims; study as a possible amplifier of hearing burden
ETD + frailty [31,32]	Frailty modifies vulnerability and management; direct ETD-frailty evidence is absent	Hypothesis-generating	Incorporate frailty into decision-making, not causal interpretation

Summary of the clinical implications in older adults driven by ETD, along with their current evidence support.

**Table 3:** Diagnostic tools for ETD and applicability in older adults.

Diagnostic Tool	What it Assesses	Strengths	Limitations	Applicability in Older Adults
Clinical history [4,9,31,32,42]	Symptoms, chronology, triggering factors, comorbidities	Widely available; identifies fluctuating or baro-challenge symptoms	Low specificity if used alone	Essential first step, distinguish pressure-triggered symptoms from nonspecific hearing/balance complaints
Otoscopy [4,9,11]	Tympanic membrane appearance and retraction/effusion	Simple and inexpensive	May be normal in intermittent/baro-challenge ETD	Useful as part of the standard office assessment
Tympanometry [4,9,11]	Middle-ear pressure and compliance	Objective and accessible	Normal results do not exclude intermittent or baro-challenge ETD	Highly useful in older adults with hearing loss or suspected conductive components; supports obstructive/dilatory phenotype when negative pressure, retraction or effusion is present
Audiometry [4,9]	Hearing thresholds and conductive/mixed components	Clarifies the interaction between ETD and presbycusis	Does not directly measure tubal function	Important to define the hearing-related burden; separates high-frequency presbycusis from potentially reversible conductive components
ETDQ-7 [9,11,21]	Patient-reported symptom burden and follow-up	Easy to administer; useful for follow-up	Insufficient diagnostic accuracy as a stand-alone diagnostic tool	Helpful as an adjunct when used with objective testing; avoid equating symptom score with confirmed obstruction
Tubomanometry/sonotubometry [17]	Dynamic tubal opening function	Provides functional information	Limited availability; requires expertise	Useful in selected or referral-center cases or diagnostic uncertainty
Nasopharyngoscopy [4,9,39]	Nasopharyngeal anatomy, inflammation, tubal orifice, masses	Direct visualization and exclusion of structural causes	More invasive than questionnaires; operator-dependent	Important in unilateral or persistent disease and in red-flag cases
CT/MRI/dynamic imaging [18,43]	Anatomical and, in selected protocols, functional information	Useful for complex, refractory, or secondary ETD	Not routine; cost and limited availability	Appropriate when malignancy, skull-base disease, or anatomical obstruction is suspected; use when secondary obstruction, skull-base disease, malignancy or complex anatomy is suspected.
Comprehensive geriatric assessment [1,5,7,8,30,31,32]	Frailty, cognition, falls risk, polypharmacy, functional reserve	Improves patient-centered decision-making	Not ET-specific; requires multidisciplinary integration	Highly relevant when choosing invasive versus conservative management.

The principal diagnostic tools for ETD and their applicability in older adults are summarized in Table 3.

**Table 4:** Therapeutic options for ETD in older adults.

Treatment Option	Main Indication	Potential Benefits	Limitations/Risks	Specific Considerations in Older Adults
Patient education and conservative measures [19,20]	Mild or intermittent symptoms; early management	Low cost; may reduce pressure-related symptoms	Variable efficacy; adherence may be limited	Useful first-line approach but instructions should be simplified when cognition or frailty is present
Management of associated upper-airway disease (rhinitis/CRS/GERD/OSA) [3,9,27,28,29,44]	Inflammatory or reversible contributors to ETD	Addresses modifiable drivers; may improve tubal ventilation and symptom burden	Benefits may be indirect and not immediate	Highly relevant because multimorbidity is common in older adults
Medical therapy (e.g., intranasal corticosteroids, selected anti-inflammatory treatment) [19,20]	Inflammatory or allergic component	May improve associated sinonasal inflammation	Evidence for ETD itself is limited; medication burden may increase	Polypharmacy and adherence should be considered carefully
Autoinflation/pressure-equalization maneuvers [19,20]	Selected patients with preserved ability to perform maneuvers	Noninvasive; may provide symptom relief	Limited efficacy in chronic obstructive ETD; rare pressure-related complications	May be difficult in cognitively impaired or frail individuals; should be recommended cautiously due to polypharmacy and adverse effects
Ventilation tube insertion [4,19,20]	Persistent middle-ear pressure dysregulation or effusion	Rapid symptom relief; bypasses impaired tubal function	Does not restore tubal function; risk of otorrhea, perforation, repeat procedures	May be appropriate when hearing rehabilitation is limited by conductive dysfunction
Balloon dilation of the Eustachian tube (BDET/BET) [14]	Selected adults with persistent obstructive ETD after failure of conservative treatment	Improves symptoms and objective findings in selected adult studies	Evidence in frail elderly patients remains limited; procedural candidacy varies	Requires individualized risk–benefit assessment including frailty, comorbidity, and life expectancy. Exclude patulous ETD
Optimization of hearing rehabilitation	ETD superimposed on presbycusis or mixed hearing loss	May improve hearing-aid benefit and communication outcomes	Does not treat the underlying tubal disorder	Particularly important in older adults because ETD may compromise the benefit of hearing devices
Multidisciplinary/geriatric-informed management	Complex or frail older adults	Promotes individualized, patient-centered care	Resource-intensive	Relevant when treatment decisions must balance symptoms, frailty, cognition, fall risk, and global functional goals

The main therapeutic options for ETD in older adults are summarized in Table 4.

## Data Availability

No new data were created or analyzed in this study. Data sharing is not applicable to this article.
